# Stiffness Variation of 3D Collagen Networks by Surface Functionalization of Network Fibrils with Sulfonated Polymers

**DOI:** 10.3390/gels7040266

**Published:** 2021-12-16

**Authors:** Philipp Riedl, Maria Schricker, Tilo Pompe

**Affiliations:** Institute of Biochemistry, Faculty of Life Sciences, Leipzig University, 04103 Leipzig, Germany; philipp.riedl@uni-leipzig.de (P.R.); ms61bipy@studserv.uni-leipzig.de (M.S.)

**Keywords:** collagen, network mechanics, sulfonated polymers

## Abstract

Fibrillar collagen is the most prominent protein in the mammalian extracellular matrix. Therefore, it is also widely used for cell culture research and clinical therapy as a biomimetic 3D scaffold. Charged biopolymers, such as sulfated glycosaminoglycans, occur in vivo in close contact with collagen fibrils, affecting many functional properties such as mechanics and binding of growth factors. For in vitro application, the functions of sulfated biopolymer decorations of fibrillar collagen materials are hardly understood. Herein, we report new results on the stiffness dependence of 3D collagen I networks by surface functionalization of the network fibrils with synthetic sulfonated polymers, namely, poly(styrene sulfonate) (PSS) and poly(vinyl sulfonate) (PVS). A non-monotonic stiffness dependence on the amount of adsorbed polymer was found for both polymers. The stiffness dependence correlated to a transition from mono- to multilayer adsorption of sulfonated polymers on the fibrils, which was most prominent for PVS. PVS mono- and multilayers caused a network stiffness change by a factor of 0.3 and 2, respectively. A charge-dependent weakening of intrafibrillar salt bridges by the adsorbed sulfonated polymers leading to fibrillar softening is discussed as the mechanism for the stiffness decrease in the monolayer regime. In contrast, multilayer adsorption can be assumed to induce interfibrillar bridging and an increase in network stiffness. Our in vitro results have a strong implication on in vivo characteristics of fibrillar collagen I, as sulfated glycosaminoglycans frequently attach to collagen fibrils in various tissues, calling for an up to now overlooked impact on matrix and tendon mechanics.

## 1. Introduction

Collagen I (Coll I) is one of the most prominent proteins of the extracellular matrix in mammalian tissues. It constitutes fibrillar networks and tendons as a very important structural component [1]. Because of this, and originating from easily applicable reconstitution approaches for fibrillar Coll I networks from rat or bovine Coll I, 3D fibrillar Coll I networks are a widely distributed gold standard for biomimetic 3D cell culture scaffolds [2,3,4].

In addition, Coll I fibrils are known to frequently occur in vivo in tight association with different sulfated glycosaminoglycans. Important functions of glycosaminoglycans are discussed for regulating Coll I fibril formation, e.g., fibromodulin, hydration degree of tissues as well as binding, presentation, and release of cytokines and growth factors [5,6,7,8,9]. These functions were also considered in matrix engineering approaches for functional Coll I networks, showing in vitro an impact of artificially sulfated hyaluronan and heparin as anti-inflammatory, pro-osteogenic, and cutaneous wound healing regulators [10,11,12,13]. Furthermore, the amount, length, and sulfation pattern of sulfated glycosaminoglycans in human skin has been shown to change over the course of intrinsic aging and photoaging, leading to varying glycosaminoglycan concentrations, charge densities, and water contents in dermal tissues [14,15,16,17,18,19].

While these biochemical impacts of sulfated glycosaminoglycans are well recognized, the possible impact on Coll I fibril mechanics has almost not been studied up to now. This is astonishing, as the viscoelastic properties of the extracellular matrix, and specifically of Coll I networks and fibrils, are heavily studied as a very important parameter and regulator of cell and tissue function [20,21]. We therefore set out to provide first insights into the impact of sulfated glycosaminoglycan association with fibrillar Coll I on the elasticity of 3D Coll I networks. Using defined 3D fibrillar Coll I networks, as a biomimetic model of the extracellular matrix of various tissues, we investigated their functionalization with different synthetic sulfonated polymers. The high degree of sulfonate groups of such polymers are good mimics of sulfated glycosaminoglycans [22]. In our study, we investigated the impact of adsorption of sulfonated polymers on Coll I fibrils in the 3D networks on the network stiffness and correlated it to the quantitative characterization of polymer adsorption on the fibrils of Coll I networks.

## 2. Results and Discussion

As stated above, our study focused on the adsorptive functionalization of Coll I networks with glycosaminoglycan-like sulfonated polymers. The quantitative analysis of ad- and desorption kinetics of the polymers was correlated to measurements of the stiffness of the 3D Coll I networks.

### 2.1. Preparation of Defined Fibrillar 3D Collagen Matrices

**Figure 1:**
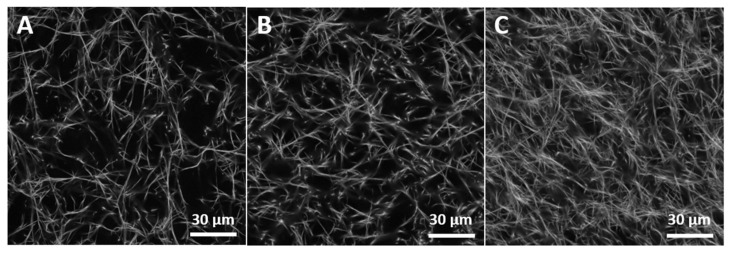
Structure and topology of collagen I matrices. (**A**–**C**): Characteristic Coll I network structure for different Coll I concentrations ((**A**): 1.5 mg/mL, (**B**): 2.0 mg/mL, (**C**): 2.5 mg/mL). Shown are laser-scanning images with a 40× magnification factor.

We used our established platform of 3D fibrillar Coll I networks with defined topology and mechanics [23,24,25]. Figure 1 shows characteristic microscopic images of such networks at varying Coll I concentrations, demonstrating options to adjust network topology. The topological parameters were determined using our previously reported image analysis tool using confocal fluorescence microscopy data [26]. In the following experiments, we used networks at a Coll I concentration of 2.5 mg/mL, exhibiting an average pore diameter of 3.35 ± 0.2 μm and a fibril diameter of 0.41 ± 0.02 μm. Stiffness of such 3D Coll I networks are in the range of 80 ± 2.6 Pa as determined by measurements of Young’s modulus using colloidal probe force spectroscopy.

### 2.2. Non-Monotonic Influence of Sulfonated Polymer Adsorption on Coll I Network Stiffness

We hypothesized that sulfated and sulfonated polymers influence Coll I network properties, as sulfated glycosaminoglycans occur in vivo in direct contact with Coll I fibrils. To mimic such surface-modification of Coll I fibrils, we used poly(styrene sulfonate) (PSS) and poly(vinyl sulfonate) (PVS) as easily available sulfated mimics of glycosaminoglycans [22]. For both polymers, a high negative charge density of deprotonated sulfonate groups can be expected at physiological conditions with pH 7. As a strong adsorptive interaction of sulfated glycosaminoglycans at physiological pH is well-known from our previous studies [24], we used a direct adsorption strategy at neutral buffer conditions for the surface functionalization of Coll I fibrils of the reconstituted 3D networks.

**Figure 2:**
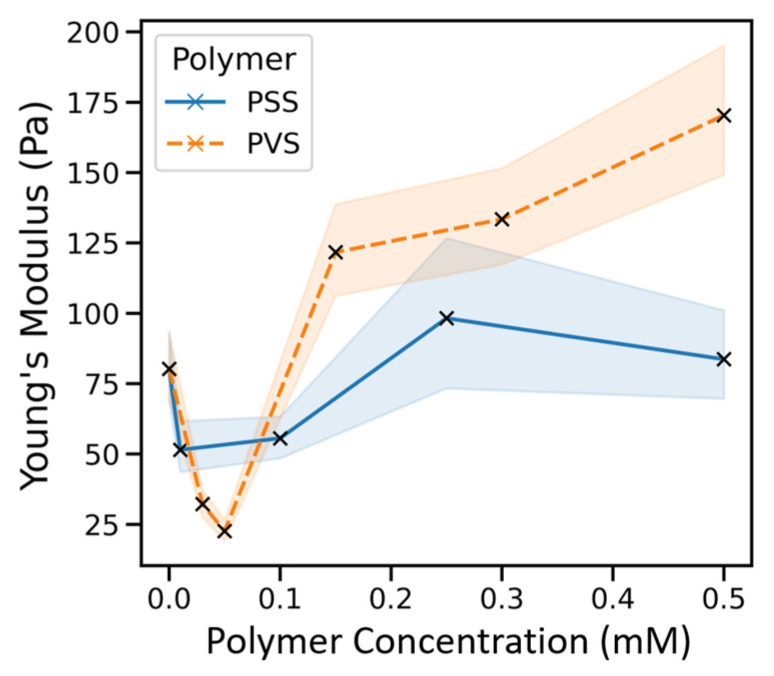
Impact of PSS and PVS functionalization on stiffness of 3D Coll I networks. Different functionalization concentrations of the polymers resulted in changes in Coll I network stiffness. Shown are medians with the highlighted confidence interval of 95%.

Using different concentrations of PSS and PVS, we adsorptively functionalized reconstituted Coll I networks and subsequently determined their Young’s modulus using colloidal probe force microscopy. We found a distinct and non-monotonic dependence of network stiffness on the polymer concentration. At low concentrations of approximately 0.05 mM, both polymers consistently led to a decreased Coll I network stiffness. However, at higher concentrations (>0.2 mM) an increase in concentration led to an increase in network stiffness (Figure 2). The mechanisms for these mechanical effects are discussed at a later stage (Section 2.5).

In conjunction with these experiments, we also checked for changes in Coll I network topology. From previous studies, we did not expect changes in pore size and fibril diameter for modification of 3D fibrillar Coll I networks [24], which we could confirm also for the experiment in the current study using the two different polymers (Figure S2).

### 2.3. Adsorption Isotherms of Sulfonate Polymers for Coll I Networks Exhibited Mono- and Multilayer Characteristics

**Figure 3:**
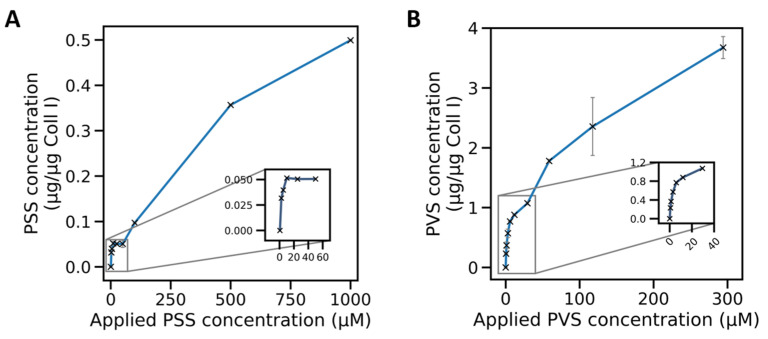
Kinetics of the adsorption of PSS and PVS on 3D Coll I matrices. The amount of adsorbed poly(styrene sulfonate) (PSS) (**A**) and poly(vinyl sulfonate) (PVS) (**B**) on fibrillar Coll I networks (normalized to the amount of Coll I in the network, approximately 100 µg) in dependence on the polymer concentration in solution.

To investigate the adsorption behavior of the model polymers, we measured the adsorbed amount of polymer per Coll I network for different functionalization concentrations as shown in Figure 3. The resulting adsorption isotherms of PSS and PVS exhibited characteristic features of mono- to multilayer transition. After a steep increase at low concentrations, a plateau phase was observed at approximately 30–50 µM, with a further increase at higher concentrations. Such a type II adsorption isotherm is typically observed for initial monolayer adsorption and transition to multilayer adsorption at higher concentrations [27,28,29]. For PSS solutions, it is known that monomer–monomer interactions and structure-formation-related viscosity increases occur at high concentrations and charge densities [30]. Counterion-mediated interchain bridging is discussed, too, suggesting a stronger tendency toward multilayer formation [31,32].

From our observations and reports in the literature, it can be concluded that at low polymer concentrations, a monolayer adsorption of sulfonated polymer coils occurs on Coll I fibrils, while at higher polymer concentrations, the polymers interact with each other leading to multilayer formation on Coll I fibrils.

### 2.4. Desorption Kinetics of PSS and PVS from Coll I Networks Indicated High Monolayer Stability

In the adsorption experiments, we observed a transition from mono- to multilayer behavior. To characterize both states, we investigated the stability of the differently adsorbed polymer layers on Coll I fibrils. It is important to receive more insight into the interaction of the sulfonated and sulfated polymers with Coll I fibrils, as we observed a concentration-dependent stiffness of the 3D Coll I networks.

**Figure 4:**
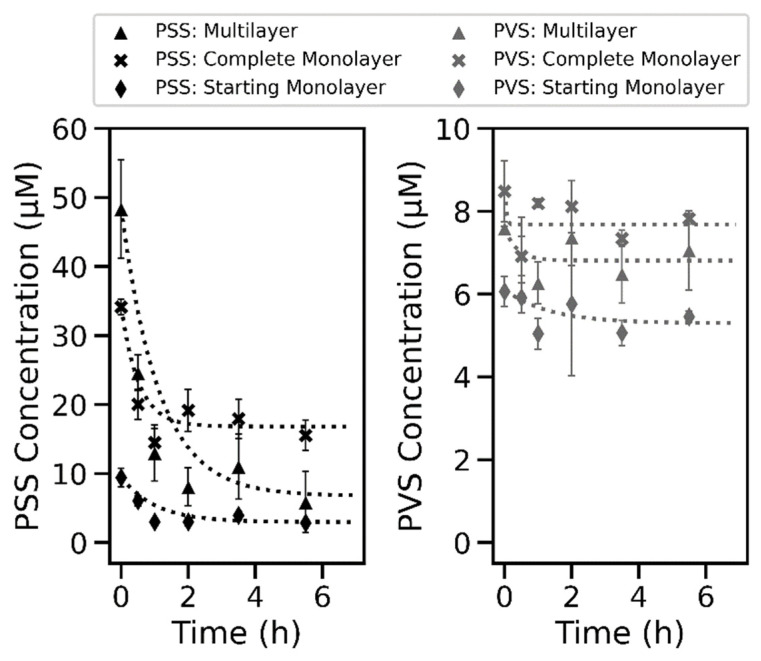
Kinetics of the desorption of PSS and PVS from 3D Coll I networks. The amount of adsorbed polymer per Coll I network was measured and normalized to initially adsorbed amounts with loading concentrations of 1.0, 0.1, and 0.01 mM for PSS and 0.3, 0.03, and 0.008 mM for PVS. Desorption kinetics were fitted mono-exponentially as indicated by the dotted lines.

In the desorption experiments, we followed the time-dependent amount of polymer adsorbed to the 3D Coll I networks (Figure 4). As starting points, we chose three different concentrations for each of the two polymers. To compare the stability of the polymer mono- and multilayers, we chose three concentrations representing the beginning of a complete monolayer plateau (“Starting Monolayer”, PSS: 10 µM, PVS: 6 µM), the transition point before entering the multilayer regime (“Complete Monolayer”, PSS: 100 µM, PVS: 30 µM), and a high concentration in the multilayer regime (“Multilayer”, PSS: 1000 µM, PVS: 300 µM). From the adsorption isotherms we expected two states for PSS and PVS. The shown polymer amounts present in the Coll I networks after the respective time durations of desorption in phosphate-buffered saline (PBS) buffer indicate differences in the stability of the adsorbed layer, which correlates to the adsorption isotherms and the mono- and multilayer states. While for PVS, almost no desorption was observed, pointing to both stable mono- and multilayer adsorption; a strong desorption (80% of initially adsorbed amount) with a time constant of 1.8 h was determined for the multilayer regime for PSS. In contrast, the PSS monolayer exhibited high stability with a time constant of 13.7 h. We can estimate the remaining amount of adsorbed PSS to be in the range of the plateau value of the adsorption experiments, indicating a high stability of the monolayer adsorption but a weaker interaction between PSS molecules in the multilayer regime.

### 2.5. Correlating Sulfonated Polymer Adsorption to Coll I Fibril Stiffness

**Figure 5:**
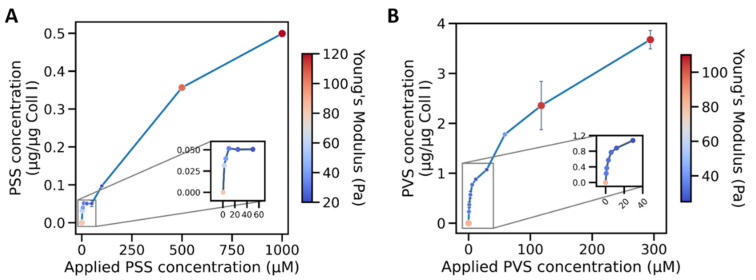
Kinetics of the adsorption of PSS and PVS on 3D Coll I matrices overlaid with respective Young’s moduli. Amount of adsorbed poly(styrene sulfonate) (PSS) (**A**) and poly(vinyl sulfonate) (PVS) (**B**) on fibrillar Coll I networks (normalized to the amount of Coll I in the network, approximately 100 µg) in dependence on the functionalization concentration. The stiffness of the corresponding matrices is shown as a color-coded overlay. Single Young’s moduli for mono- & multilayer states are compared with unfunctionalized Coll I networks in Figure S1.

In the context of the different mono- and multilayer states of sulfonated polymer adsorption on Coll I fibrils in the 3D networks, it is now interesting to discuss the measured network stiffness. Figure 5 shows a correlation diagram of network stiffness and the amount of adsorbed polymer. One can clearly see that the decrease in network stiffness correlated with the monolayer adsorption of sulfonated polymers. The observed increase in network stiffness for higher polymer solution concentrations can be correlated to the multilayer adsorption of the polymers.

Hence, we can discuss two adsorption regimes influencing the network stiffness of fibrillar 3D Coll I networks. As expected, the respective regimes of the two polymers were found to be set apart concerning polymer solution concentration by a similar factor as their molecular weights. This fact indicates that the molecular weight (size) of the polymer influences the critical concentration of mono- and multilayer formation.

For monolayer adsorption and tight interactions of the sulfonated polymer molecules with Coll I fibrils, a softening of the fibrils has to be considered. Network stiffness in fibrillar Coll I networks with quite thick fibrils is usually discussed as being bending-dominated, controlled by the bending stiffness of the Coll I fibrils [33,34]. As we did not observe any changes in network topology (see above), the change in network stiffness has to be attributed to changes in the bending modulus of the Coll I fibrils.

Previous studies on structure, stability, and mechanics of Coll I fibrils in dependence on ionic strength of buffer solutions and osmotic pressure by charged groups [35,36,37] provide arguments for the direct influence of highly charged polymers adsorbed on fibril surfaces on the intrafibrillar structure and mechanics. It is known that a decrease in ionic strength of the surrounding buffers leads to a weakening of intrafibrillar salt bridges of Coll I fibrils and a less ordered intrafibrillar structure of tropocollagen molecules in the fibrils. These parameters are strongly related to the mechanical properties, such as bending modulus, of Coll I fibrils [38]. Furthermore, charge-overcompensation on Coll I fibrils by Ca^2+^ ions is also known to weaken intrafibrillar interaction by binding to negatively charged carboxylate moieties, possibly affecting charged amino acid side chains for salt bridge formation [35]. Now, adsorption of highly negatively charged sulfonated and sulfate polymers on Coll I fibril surfaces can be expected to lead to a strong influence on local electrostatic interaction and a weakening of intrafibrillar salt bridges within the Coll I fibrils. Positively charged lysine and arginine side chains of tropocollagen molecules can be expected to be inhibited to contribute to intrafibrillar crosslinking and salt bridge formation by the presence of the negatively charged adsorbed polymers. Our desorption studies also imply that this inhibition by the adsorbed sulfonated polymers is very stable as extensive rinsing in buffer solution do not allow to desorb the strongly interacting monolayer. The discussed behavior also fits with the previously reported stiffening and structural influence of an increased osmotic pressure on Coll I fibrils [39]. An increased local hydration of Coll I fibrils by the high surface charge density of the adsorbed polymers could, in theory, lead to a decrease in osmotic pressure inside the Coll I fibrils and, again, to the discussed loosening of the intrafibrillar structure and softening of the fibrils.

From this discussion, we conclude that an adsorption of highly negatively charged polymers on Coll I fibrils influences the intrafibrillar structure and stability of salt bridges, leading to a decrease in the bending stiffness and a resulting decrease in Coll I network stiffness. The contrasting behavior of a reversal of the observed softening and even increase in network stiffness for high polymer concentrations has to be attributed to the multilayer adsorption regime and interfibrillar crosslinking of the 3D network. It is well documented that electrostatic polymer–polymer interactions of PSS at high concentrations lead to highly viscous solutions; hence, such interactions should also be present here in the multilayer adsorption regime. Multivalent counterion-induced bridging interactions have been simulated and described for carboxylate polyelectrolytes, showing strong polymer–polymer interactions mediated especially by monovalent counterions such as the Na^+^ ions in our polymer solutions [31]. Chialvo and Simonson could also demonstrate evidence for like-charge attraction among sulfonate groups of PSS [40]. It was shown that while counterions mediate interchain bridges among sulfonate groups, intrachain sulfonate interactions are mediated by water molecules. Furthermore, PSS in aqueous solutions was found to exhibit an increasing stiffness with increasing polymer solution concentration, mainly caused by the osmotic pressure of the sodium counterions [30]. Taken together, one can argue that the high concentrations of PSS (and PVS) in our experiments led to a structural stiffening of the functionalized matrix due to the bridging interactions between polymer molecules.

## 3. Conclusions

In this study, we demonstrated the distinct influence of the sulfonated polymers PSS and PVS in dependence on their solution concentration during adsorption on fibrillar Coll I networks. Thereby, the Coll I networks exhibited a non-monotonic stiffness dependence on the amount of adsorbed polymer. The adsorption kinetics of these polymers indicated two different adsorption regimes, monolayer and multilayer, leading to a decrease or an increase in the stiffness of the Coll I networks, respectively. The results suggest a destabilizing effect of highly charged sulfate and sulfonate polymers when bound to Coll I fibrils by influencing intrafibrillar salt bridges. Furthermore, interfibrillar bridging was discussed as a mechanism for the increase in network stiffness in the multilayer adsorption regime at high polymer concentrations. Sulfated glycosaminoglycans are often found in vivo to be associated with Coll I fibrils at different densities and compositions. Hence, our findings implicate a control mechanism of tissue and extracellular matrix stiffness composed of Coll I fibrils, which has been, up to now, overlooked in cell biology, biomedicine, and biomaterials research. Furthermore, we propose the usage of synthetic and natural sulfated and sulfonated polymers as a relevant tool to engineer and control the mechanics of fibrillar 3D Coll I networks for matrix and tissue engineering approaches.

## 4. Materials and Methods

### 4.1. Reconstitution of Collagen I Networks

Coll I networks were reconstituted on 13 mm glass coverslip coated with 0.14% *w/w* poly(styrene-*alt*-maleic anhydride) (PSMA; MW 30,000 g/mol; Sigma–Aldrich, Taufkirchen, Germany) according to previous reports [41]. All Coll I preparation steps prior to matrix reconstitution were performed on ice. Subsequently, the mixtures were transferred onto a glass coverslip. Coll I stock solution (3.04 mg/mL (rat tail, Corning, New York, NY, USA)) was prediluted in acetic acid (0.02 M) and phosphate buffer (0.25 M). The final Coll I reconstitution solution had a pH of 7.5. A total volume of 40 µL of the Coll I reconstitution solution was placed on PSMA-coated glass coverslips, and the Coll I fibril formation was immediately initiated by transfer to 37 °C (95% relative humidity, 5% CO_2_) for 40 min. Fibrillated Coll I networks were rinsed 3 times with phosphate-buffered saline (PBS, Biochrom, Berlin, Germany) prior to analysis and functionalization. After reconstitution, the networks exhibited an overall network thickness of roughly 350 µm.

### 4.2. Polymer Functionalization of Collagen I Networks

Poly(vinly sulfonate) sodium (PVS, Mw ∼170 kD) and poly(styrene sulfonate) sodium (PSS, Mw ∼70 kD) were purchased from Sigma–Aldrich Chemical Co. (Taufkirchen, Germany), and were used without further purification. The respective polymers were dissolved at appropriate concentrations in PBS (1×) buffer, and 500 μL of the solution was placed into wells of a 24-well plate. The Coll I networks on the glass coverslips were immersed into the polymer solution and incubated at 37 °C for 1 h. The polymer solution was then removed, and the networks were washed twice with PBS (1×) buffer. For the functionalization, different concentrations of PSS and PVS were applied to account for the difference in their molecular weights.

### 4.3. Desorption Measurements

Coll I networks were functionalized with three different concentrations of PSS (1.0, 0.1, and 0.01 mM) or PVS (0.3, 0.03, and 0.006 mM) as described, representing an emerging monolayer, a completed monolayer, and a multilayer regime, respectively. After rinsing the Coll I networks three times with PBS (1×), the decrease in the amount of adsorbed polymer was measured over the course of 5.5 h at room temperature using Toluidine blue measurements (see Section 4.5).

### 4.4. Adsorption Measurements

Coll I networks were functionalized with a range of concentrations of PSS or PVS. Subsequently, the Coll I networks were rinsed three times with PBS (1×) and the remaining amount of stably adsorbed polymer was determined at room temperature using the Toluidine blue assay (see Section 4.5). For the polymer functionalization, we used the following concentrations: PSS: 0.5, 2.5, 5, 10, 25, 50, 100, 500, and 1000 µM; PVS: 0.6, 1, 3, 6, 12, 30, 60, 120, and 300 µM. 

### 4.5. Toluidine Blue Assay

The functionalized Coll I networks were digested for 2 h at 37 °C using 200 µL of a 200 U/mL collagenase IV solution. Afterwards, 100 µL of the solution was transferred to a 96-well plate and 100 µL Toluidine blue (0.1 mM) and 100 µL PBS was added. Using a photometer, the absorbance of Toluidine blue between 550 and 680 nm was measured in the well plate. Using the absorbance shift and the peak intensity at 642 nm, the PSS concentration was calculated from respective calibration curves.

### 4.6. Colloidal Probe Force Spectroscopy

The elastic modulus of the reconstituted Coll I networks was determined by colloidal probe force spectroscopy using a scanning force microscope (NanoWizard 3, JPK Instruments, Berlin, Germany), as previously reported [34]. Briefly, a 50 μm glass microbead (Polyscience Europe GmbH, Eppelheim, Germany) was attached to a tipless HQ-CSC38 cantilever (NanoAndMore, Wetzlar, Germany) with a spring constant of ∼0.1 N/m. Exact spring constant was determined by the thermal noise method [42]. A measurement of at least 50 force–distance curves at 3 positions of each Coll I matrix with 3 independent experiments was conducted in PBS buffer (Biochrom, Berlin, Germany) at room temperature. Indentation rate was set to 5 μm/s. As a measure of network elasticity, we used the Young’s modulus as determined by fitting the retract part of force distance curves (typical indentation depth 5 μm) using the Hertz model, see also [25]. Repetitive measurements at the same position indicated no viscoplastic behavior, as they resulted in similar force–distance curves. For the polymer functionalization, we used the following concentrations: PSS: 0.01, 0.1, 0.25, and 0.5 mM; PVS: 0.03, 0.05, 0.15, 0.3, and 0.5 mM.

### 4.7. Statistical Analysis

Experiments were performed at least in triplicate, if not stated otherwise. Error bars indicate standard deviation (SD), if not stated otherwise.

## Data Availability

The data sets generated and analyzed during the current study are available from the corresponding author upon reasonable request.

## Supplementary Materials

The following are available online at https://www.mdpi.com/article/10.3390/gels7040266/s1, Figure S1: Impact of PSS and PVS functionalization on stiffness of 3D Coll I networks, Figure S2: Impact of PSS and PVS functionalization on the topology of 3D Coll I networks.

## References

[1] Naomi R., Ridzuan P.M., Bahari H. (2021). Current Insights into Collagen Type I. Polymers.

[2] Chowdhury S.R., Mh Busra M.F., Lokanathan Y., Ng M.H., Law J.X., Cletus U.C., Binti Haji Idrus R., Chun H.J., Park K., Kim C.-H., Khang G. (2018). Collagen Type I: A Versatile Biomaterial. Novel Biomaterials for Regenerative Medicine.

[3] Lee C.H., Singla A., Lee Y. (2001). Biomedical applications of collagen. Int. J. Pharm..

[4] Sapudom J., Pompe T. (2018). Biomimetic tumor microenvironments based on collagen matrices. Biomater. Sci..

[5] Scott R.A., Panitch A. (2013). Glycosaminoglycans in biomedicine. Wiley Interdiscip. Rev. Nanomed. Nanobiotechnol..

[6] Freudenberg U., Liang Y., Kiick K.L., Werner C. (2016). Glycosaminoglycan-Based Biohybrid Hydrogels: A Sweet and Smart Choice for Multifunctional Biomaterials. Adv. Mater..

[7] Pourhanifeh M.H., Mohammadi R., Noruzi S., Hosseini S.A., Fanoudi S., Mohamadi Y., Hashemzehi M., Asemi Z., Mirzaei H.R., Salarinia R. (2019). The role of fibromodulin in cancer pathogenesis: Implications for diagnosis and therapy. Cancer Cell Int..

[8] Jan A.T., Lee E.J., Choi I. (2016). Fibromodulin: A regulatory molecule maintaining cellular architecture for normal cellular function. Int. J. Biochem. Cell Biol..

[9] Parry D.A.D., Flint M.H., Gillard G.C., Craig A.S. (1982). A role for glycosaminoglycans in the development of collagen fibrils. FEBS Lett..

[10] Hempel U., Matthäus C., Preissler C., Möller S., Hintze V., Dieter P. (2014). Artificial Matrices With High-Sulfated Glycosaminoglycans and Collagen Are Anti-Inflammatory and Pro-Osteogenic for Human Mesenchymal Stromal Cells. J. Cell. Biochem..

[11] Salbach-Hirsch J., Ziegler N., Thiele S., Moeller S., Schnabelrauch M., Hintze V., Scharnweber D., Rauner M., Hofbauer L.C. (2014). Sulfated Glycosaminoglycans Support Osteoblast Functions and Concurrently Suppress Osteoclasts. J. Cell. Biochem..

[12] Friedemann M., Kalbitzer L., Franz S., Moeller S., Schnabelrauch M., Simon J.-C., Pompe T., Franke K. (2017). Instructing Human Macrophage Polarization by Stiffness and Glycosaminoglycan Functionalization in 3D Collagen Networks. Adv. Healthc. Mater..

[13] Anderegg U., Halfter N., Schnabelrauch M., Hintze V. (2021). Collagen/glycosaminoglycan-based matrices for controlling skin cell responses. Biol. Chem..

[14] Carrino D.A., Calabro A., Darr A.B., Dours-Zimmermann M.T., Sandy J.D., Zimmermann D.R., Sorrell J.M., Hascall V.C., Caplan A.I. (2011). Age-related differences in human skin proteoglycans. Glycobiology.

[15] Wang S.T., Neo B.H., Betts R.J. (2021). Glycosaminoglycans: Sweet as Sugar Targets for Topical Skin Anti-Aging. Clin. Cosmet. Investig. Dermatol..

[16] Oh J.-H., Kim Y.K., Jung J.-Y., Shin J., Kim K.H., Cho K.H., Eun H.C., Chung J.H. (2011). Intrinsic aging- and photoaging-dependent level changes of glycosaminoglycans and their correlation with water content in human skin. J. Dermatol. Sci..

[17] Lee D.H., Oh J.-H., Chung J.H. (2016). Glycosaminoglycan and proteoglycan in skin aging. J. Dermatol. Sci..

[18] Bernstein E.F., Underhill C.B., Hahn P.J., Brown D.B., Uitto J. (1996). Chronic sun exposure alters both the content and distribution of dermal glycosaminoglycans. Br. J. Dermatol..

[19] Oh J.-H., Shin M.K., Lee H., Lim J., Choi M., Cho S., Chung J.H. (2018). Analysis of sulfated glycosaminoglycan composition change in intrinsically aged and photoaged human skin using an enzymatic degradation method. J. Dermatol. Sci..

[20] Chaudhuri O., Cooper-White J., Janmey P.A., Mooney D.J., Shenoy V.B. (2020). Effects of extracellular matrix viscoelasticity on cellular behaviour. Nature.

[21] Elosegui-Artola A. (2021). The extracellular matrix viscoelasticity as a regulator of cell and tissue dynamics. Curr. Opin. Cell Biol..

[22] Place E.S., Evans N.D., Stevens M.M. (2009). Complexity in biomaterials for tissue engineering. Nat. Mater..

[23] Kalbitzer L., Pompe T. (2018). Fibril growth kinetics link buffer conditions and topology of 3D collagen I networks. Acta Biomater..

[24] Kalbitzer L., Franke K., Möller S., Schnabelrauch M., Pompe T. (2015). Glycosaminoglycan functionalization of mechanically and topologically defined collagen I matrices. J. Mater. Chem. B.

[25] Sapudom J., Rubner S., Martin S., Kurth T., Riedel S., Mierke C.T., Pompe T. (2015). The phenotype of cancer cell invasion controlled by fibril diameter and pore size of 3D collagen networks. Biomaterials.

[26] Franke K., Sapudom J., Kalbitzer L., Anderegg U., Pompe T. (2014). Topologically defined composites of collagen types I and V as in vitro cell culture scaffolds. Acta Biomater..

[27] Sing K.S.W. (1985). Reporting physisorption data for gas/solid systems with special reference to the determination of surface area and porosity (Recommendations 1984). Pure Appl. Chem..

[28] Hattori Y., Kaneko K., Ohba T. (2013). Adsorption Properties. Comprehensive Inorganic Chemistry II.

[29] Skalny J., Hearn N. (2001). Surface Area Measurements. Handbook of Analytical Techniques in Concrete Science and Technology.

[30] Qu D., Baigl D., Williams C.E., Möhwald H., Fery A. (2003). Dependence of Structural Forces in Polyelectrolyte Solutions on Charge Density: A Combined AFM/SAXS Study. Macromolecules.

[31] Pial T.H., Sachar H.S., Das S. (2021). Quantification of Mono- and Multivalent Counterion-Mediated Bridging in Polyelectrolyte Brushes. Macromolecules.

[32] Yu J., Jackson N.E., Xu X., Brettmann B.K., Ruths M., de Pablo J.J., Tirrell M. (2017). Multivalent ions induce lateral structural inhomogeneities in polyelectrolyte brushes. Sci. Adv..

[33] Broedersz C.P., MacKintosh F.C. (2014). Modeling semiflexible polymer networks. Rev. Mod. Phys..

[34] Sapudom J., Kalbitzer L., Wu X., Martin S., Kroy K., Pompe T. (2019). Fibril bending stiffness of 3D collagen matrices instructs spreading and clustering of invasive and non-invasive breast cancer cells. Biomaterials.

[35] Freudenberg U., Behrens S.H., Welzel P.B., Müller M., Grimmer M., Salchert K., Taeger T., Schmidt K., Pompe W., Werner C. (2007). Electrostatic Interactions Modulate the Conformation of Collagen I. Biophys. J..

[36] Bertinetti L., Masic A., Schuetz R., Barbetta A., Seidt B., Wagermaier W., Fratzl P. (2015). Osmotically driven tensile stress in collagen-based mineralized tissues. J. Mech. Behav. Biomed. Mater..

[37] Reich S., Katchalsky A., Oplatka A. (1968). Dynamic-elastic investigation of the chemical denaturation of collagen fibers. Biopolymers.

[38] Buehler M.J. (2006). Nature designs tough collagen: Explaining the nanostructure of collagen fibrils. Proc. Natl. Acad. Sci. USA.

[39] Masic A., Bertinetti L., Schuetz R., Chang S.-W., Metzger T.H., Buehler M.J., Fratzl P. (2015). Osmotic pressure induced tensile forces in tendon collagen. Nat. Commun..

[40] Chialvo A.A., Simonson J.M. (2005). Solvation Behavior of Short-Chain Polystyrene Sulfonate in Aqueous Electrolyte Solutions: A Molecular Dynamics Study. J. Phys. Chem. B.

[41] Stamov D.R., Pompe T. (2012). Structure and function of ECM-inspired composite collagen type I scaffolds. Soft Matter.

[42] Hutter J.L., Bechhoefer J. (1993). Calibration of atomic-force microscope tips. Rev. Sci. Instrum..

