# Supplementary material for: Stiffness Variation of 3D Collagen Networks by Surface Functionalization of Network Fibrils with Sulfonated Polymers
Source: Gels. 2021 Dec 16;7(4):266. doi: 10.3390/gels7040266 (PMC8702206; doi:10.3390/gels7040266)
Supplement: Supplementary file 1 [file gels-07-00266-s001.zip › gels-1470984-supplementary.pdf]

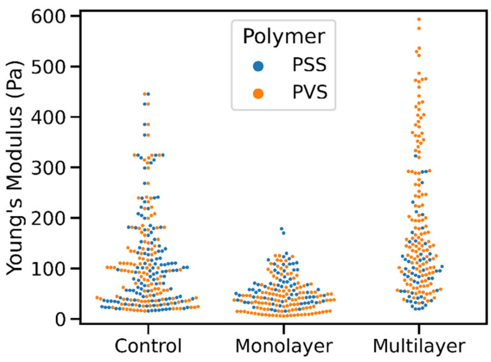

Figure S1. Impact of PSS and PVS functionalization on stiffness of 3D Coll I networks. Different concentration regimes of the polymers lead to either mono- or multilayer adsorption, resulting in a de- or increase in Coll I network stiffness, respectively. Data are shown as swarm plot from at least 25 single force spectroscopy measurements per sample on at least 3 independent samples.

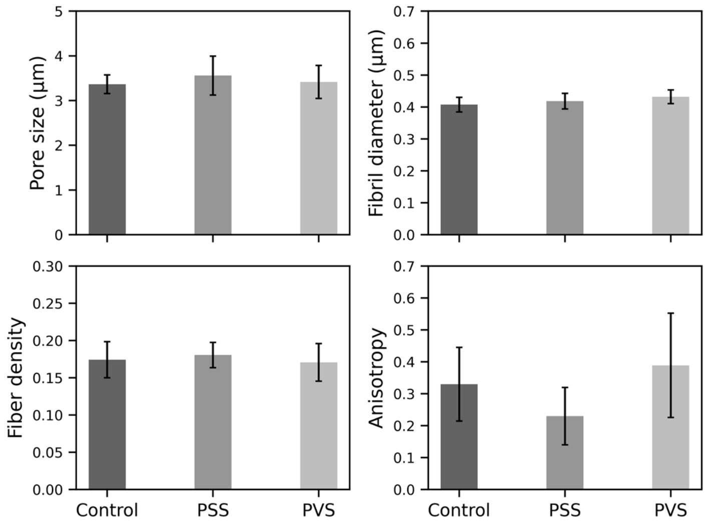

Figure S2. Impact of PSS and PVS functionalization on the topology of 3D Coll I networks. Shown are the non-significant changes in pore size, fibril diameter, fiber density and anisotropy on the overall network topology upon functionalization with PSS and PVS, compared to non-functionalized Coll I networks ("control").
